# Programmatic approaches to screening for tuberculosis disease: a situational analysis of seven countries in the Western Pacific Region

**DOI:** 10.1186/s41182-025-00846-x

**Published:** 2025-12-12

**Authors:** Alvin Kuo Jing Teo, Kyung Hyun Oh, Manami Yanagawa, Cecily Miller, Dennis Falzon, Avinash Kanchar, Youngeun Choi, Gyeong In Lee, Fukushi Morishita, Kalpeshsinh Rahevar, Rajendra Prasad Hubraj Yadav, Huong Thi Giang Tran, Regional TB Consortium, Anousone Sisouvanh, Bunleng Bith, Clarissa Blanca, Ganpurev Byambaa, Herolyn Nindil, Huyen Thanh Truong, Janet Ibay, Kim Eam Khun, Narantuya Jadambaa, Quang Hieu Vu, Sakhone Suthepmany, Serongkea Deng, Uyanga Erdenebileg, Vibol Iem, Vilath Seevisay, Walley Ambano, Zhenhong Li, Zhongdan Chen

**Affiliations:** 1 Faculty of Medicine and Health, University of Sydney, Sydney, NSW Australia; 2 The University of Sydney Infectious Diseases Institute (Sydney ID), Sydney, NSW Australia; 3 Saw Swee Hock School of Public Health, National University of Singapore, National University Health System, Singapore, Singapore; 4 World Health Organization Regional Office for the Western Pacific, Manila, the Philippines; 5 World Health Organization, Global Programme On Tuberculosis & Lung Health, Geneva, Switzerland; 6 The Korean Institute of Tuberculosis, Korean National Tuberculosis Association, Osong, Republic of Korea

## Abstract

**Background:**

Tuberculosis (TB) remains a significant public health challenge in the Western Pacific Region, which accounts for approximately 20% of the global TB burden. Despite effective diagnostic tools and treatment, many individuals with TB remain undiagnosed or unreported, particularly in high-burden countries. Systematic screening is a key strategy for identifying cases early and reducing transmission. This study presents a situational analysis of TB screening policies, practices, and challenges across seven high-burden countries in the region: Cambodia, China, Lao PDR, Mongolia, Papua New Guinea, the Philippines, and Viet Nam.

**Main body:**

Data were collected through questionnaires, follow-up discussions, and a regional workshop involving National TB Programme representatives and WHO staff. Most countries have national guidelines for systematic screening, prioritising high-risk groups, like people living with HIV and household contacts. Common screening tools include symptom screening, chest X-rays, and WHO-recommended rapid molecular diagnostics. Although asymptomatic TB is increasingly recognised, symptom screening remains the primary initial tool. Chest X-rays with computer-aided detection technologies are available in most countries, but are often limited to donor-funded projects.

Screening is conducted through routine healthcare visits, scheduled checks for specific populations (e.g., prisoners, older adults), and ad hoc campaigns. Implementation varies due to resource and infrastructure limitations. While integration with other health services and community-based approaches shows promise, these remain underutilised. Key challenges include limited funding, workforce shortages, low provider awareness, and stigma. The COVID-19 pandemic disrupted TB services, underscoring the need for resilient health systems.

**Conclusion:**

Improving systematic TB screening requires scaling up sensitive diagnostic tools, decentralising implementation, and strengthening community engagement. Sustainable financing, robust health systems, and multi-sectoral collaboration are critical to reaching the “missing millions” and achieving the End TB goals. This analysis underscores the need for targeted, evidence-based strategies to enhance screening coverage and effectiveness across diverse epidemiological and resource settings.

## Background

Tuberculosis (TB) is a significant public health concern in the Western Pacific Region, which includes 37 countries and areas with diverse economic status and TB epidemiology [1]. According to the World Health Organization (WHO), the region accounted for about 20% of the global TB burden, with an estimated 1.9 million new cases and 95,000 deaths in 2023 [2]. TB screening is a vital first step in identifying cases early, preventing transmission, and initiating timely treatment. WHO recommends targeted screening for high-risk groups, including close contacts, people living with HIV, incarcerated individuals, populations in high-incidence areas, and those with structural or clinical risk factors [3]. Screening tools include chest X-rays (CXR) and WHO-endorsed rapid molecular diagnostics. Historical evidence highlighted the effectiveness of mass CXR screening in reducing TB incidence [4] and recent advances, such as digital CXR and computer-aided detection (CAD), have renewed interest in CXR as a scalable, efficient screening tool, particularly in high-burden settings.

**Fig. 1:**
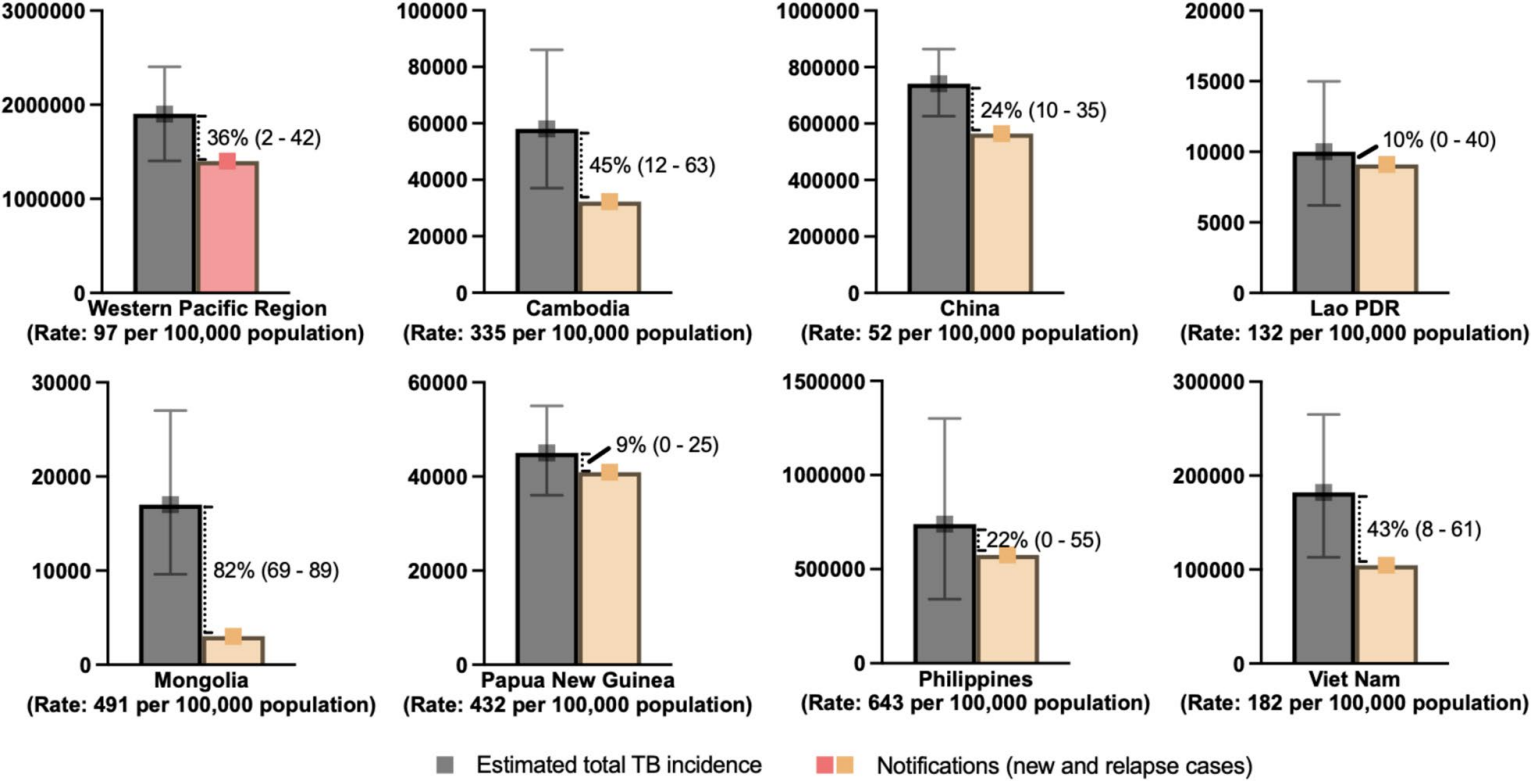
Estimated total TB incidence, case notifications, and estimated proportion of undetected/unreported cases in seven countries in the Western Pacific Region. Data were obtained from the WHO Global TB Report 2024 (country profiles). The dotted lines represent the “missing” people with TB—the difference between the estimated total TB incidence and case notification in 2023. The range in parentheses represents the estimated low and high bounds, expressed as a percentage. The rate represents the number of cases per 100,000 people per year

The Western Pacific Regional Framework to End TB identified 10 TB priority countries in the region [5]. This study focused on seven high-burden countries based on the absolute number of cases and incidence rates—Cambodia, the People’s Republic of China (hereinafter China), Lao People's Democratic Republic (hereinafter Lao PDR), Mongolia, Papua New Guinea, the Philippines, and Viet Nam (Fig. 1). China had the highest estimated TB incidence with 741,000 cases (uncertainty intervals [UI] 626,000–864000), followed by the Philippines with 739,000 (UI 340000–1280000), and Viet Nam with 182,000 (UI 113000–265000) [2]

In 2023, Mongolia showed the largest gap between notification and the best estimate of TB incidence and notified cases (82% [low bound: 69%–high bound 89%]) [2] while the Philippines, Viet Nam, and Cambodia had gaps around 36% (2–42%) [2]. Papua New Guinea and Lao PDR had the smallest estimated gap, at 9% (0–25%) and 10% (0–40%), respectively [2]. These gaps reflected both underreporting and undiagnosed TB, influenced by the strength of surveillance systems and the quality of notification data. Individuals seeking care outside national systems may go unreported [6, 7].

Although major drivers for the gap between notification rates and the estimated incidence were not well documented, undiagnosed individuals posed significant challenges for both individual and public health. This study aimed to (1) assess the TB screening policies, practices, and challenges in seven high-burden countries in the Western Pacific Region, and (2) identify potential strategies to improve coverage and effectiveness in detecting TB.

## Data sources

Data for the situational analysis were collected through various methods. Initially, a detailed questionnaire was distributed in July 2023 to WHO country focal points for TB and National TB Programme representatives, also co-authors of this paper. The questionnaire sought information on populations eligible for systematic screening, the tools and algorithms used for screening, and the TB screening strategies currently in place in each country. Follow-up online discussions were held with the country representatives to elaborate on the survey responses. Finally, a regional workshop was held from 25 to 27 October 2023 in Seoul, Republic of Korea. The event brought together representatives from National TB Programmes along with TB experts from WHO country offices, the Western Pacific Regional Office, and the WHO headquarters. The workshop aimed to discuss the findings and implementation challenges and develop country action plans. Moreover, the country incidence estimates cited and discussed in this article are derived from those produced by the WHO and published in the Global TB Report [2] A key limitation of this study was its reliance on self-reported information by NTP informants and WHO staff, without external validation through site visits or records verification. It is noteworthy that no patient-level or identifiable data were collected for this descriptive programmatic review.

## Main body: findings and discussion

### National guidelines and the eligible populations for the systematic screening for TB

All surveyed countries, except Papua New Guinea, had developed their own national guidelines for systematic TB screening. The criteria for determining eligibility for systematic TB screening varied from country to country. However, two key population groups were consistently prioritised for systematic screening: people living with HIV and household contacts of persons with bacteriologically confirmed TB (Table 1). The Philippines and Viet Nam adopted a more comprehensive approach by including all population groups recommended by the WHO in their screening guidelines.

**Table 1:**
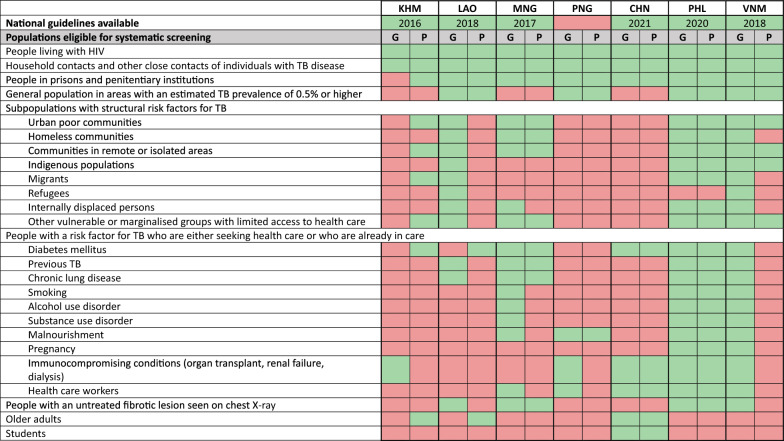
Populations eligible for systematic screening according to national guidelines (if available) in seven countries

Green cells indicate populations that are eligible for systematic screening according to national guidelines or current practice, while red cells indicate populations that are not considered eligible. The populations listed in this table are those recommended by WHO for systematic screening, except for older adults and students

*TB* tuberculosis, *HIV* human immunodeficiency virus, *G* national guidelines, *P* general practice in the country. Country abbreviations include *KHM* (Cambodia), *LAO* (Lao PDR), *MGN* (Mongolia), *PNG* (Papua New Guinea), *CHN* (People’s Republic of China), PHL (the Philippines), and *VNM* (Viet Nam)

In practice, some countries screened additional groups not formally included in their guidelines. For example, Cambodia systematically screened the urban poor, residents of remote areas, migrants, and individuals with diabetes for TB to various degrees based on evolving evidence and updated recommendations [8–10]

### Screening tools and algorithms

**Fig. 2:**
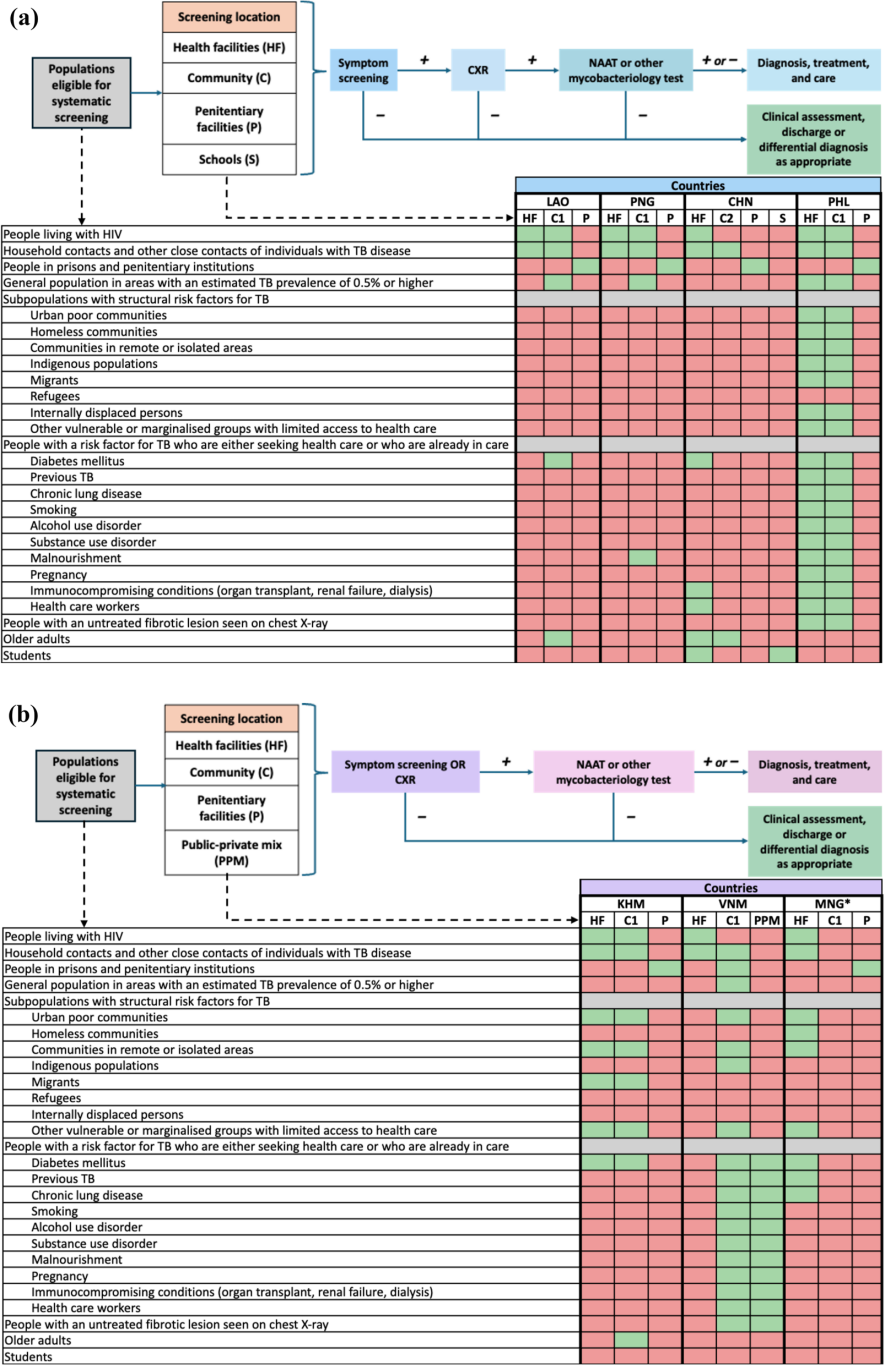
TB screening algorithms used for populations eligible for systematic screening. **(a)** Populations eligible for systematic screening in countries using symptom screening as the initial screening tool. This figure presents the populations recommended for systematic TB screening in countries that use symptom screening as the initial tool. The populations listed in this figure are all those recommended by the WHO for systematic screening, except for older adults and students. Green cells indicate populations eligible for systematic screening in the respective country, while red cells indicate those that are not. Screening locations are abbreviated as *HF* (health facilities), *C* (community), *P* (penitentiary facilities), and *S* (schools). Community-based screening is further categorised as C1 (ad hoc community-based active case finding activities) and C2 (regular community-based active case finding activities). Dashed lines link each population group to its corresponding screening location. "Mycobacteriology test" refers to nucleic acid amplification tests (NAAT); however, in some settings, smear microscopy is used due to limited access to NAAT. Country abbreviations include *LAO* (Lao PDR), *PNG* (Papua New Guinea), *CHN* (People’s Republic of China), and *PHL* (the Philippines). Symbols: “ + ” indicates positive; “–” indicates negative. (**b**) Populations eligible for systematic screening in countries using symptom screening or chest X-ray as the initial screening tool. This figure presents the populations recommended for systematic TB screening in countries that use symptom screening or chest X-ray as the initial tool. Mongolia (*) performs chest X-ray regardless of symptom status. The populations listed in this figure are all those recommended by the WHO for systematic screening, except for older adults and students. Green cells indicate populations eligible for systematic screening in the respective country, while red cells indicate those that are not. Screening locations are abbreviated as *HF* (health facilities), *C* (community), *P* (penitentiary facilities), and *PPM* (public–private mix). Community-based screening is further categorised as C1 (ad hoc community-based active case finding activities). Dashed lines link each population group to its corresponding screening location. "Mycobacteriology test" refers to nucleic acid amplification tests (NAAT); however, in some settings, smear microscopy is used due to limited access to NAAT. Country abbreviations include *KHM* (Cambodia), *VNM* (Viet Nam), and *MNG* (Mongolia). Symbols: “ + ” indicates positive; “–” indicates negative

Symptom screening was widely used as the initial screening tool (Fig. 2A and B). Individuals with symptoms typically underwent further testing, including CXR to detect abnormalities. If CXR findings suggested TB, sputum samples were tested using WHO-approved rapid molecular diagnostics (mWRD) like Xpert MTB/RIF [3]. Symptom screening was often used due to limited access to CXR or mWRD. In Mongolia (Fig. 2B), CXR was the primary screening tool for TB, regardless of symptoms [11]. In Viet Nam, CXR and symptom screening were recommended for households and close contacts of people with TB, and other high-risk groups, such as people with diabetes, people living with HIV, smokers, and people who are malnourished. In areas with limited CXR access, such as parts of Viet Nam and Cambodia, sputum testing was used when symptoms were reported [12]

CAD technologies were available in six of the seven surveyed countries, except Lao PDR. However, their use was limited and varied by software and implementation approach. CAD was mostly deployed in donor-funded project sites and often used alongside human readers rather than as a standalone tool [13]. There was no consistent data on standardised CAD use across settings. CAD systems required calibration to optimise the threshold values for their specific application context. Typically, calibration support was provided remotely by the software developers. While they guide the establishment of CAD threshold values, surveyed countries have adapted these thresholds and tailored them to local epidemiology [13, 14] For example, in the Philippines, CAD thresholds were informed by research and field data, and finalised through a national deliberative process led by the National TB Programme Task Force. This ensured alignment with both local expertise and international best practices.

### Implementation approaches

Systematic screening of at-risk groups, involving symptom assessment, imaging tools, and rapid diagnostic tests, was implemented in three main ways—(1) routine screening at every encounter; (2) screening at regular pre-determined intervals; and (3) ad hoc screening, which occurred sporadically, often in response to immediate needs, such as outbreaks or the availability of funding and resources. Across these measures, systematic screening was understood and used interchangeably with TB active case finding (ACF).

#### Systematic screening at every encounter

All countries surveyed conducted TB screening at every healthcare visit for people living with HIV. In China, this also included those with immunocompromising conditions admitted to hospitals who were assessed using symptom checks and CXR evaluations [15] However, implementing a systematic and routine approach often faced significant resource challenges. As a result, countries often adopted a more flexible approach, combining regular and ad hoc screening based on available resources and target populations.

#### Regular screening

Unlike systematic screening involving assessment at every healthcare visit, regular screening was conducted at fixed intervals. Across the assessed countries, regular TB screening was prioritised for specific populations: incarcerated individuals, close contacts of people with TB, older adults, and miners. In China, Mongolia, and Viet Nam, incarcerated persons, including staff (Mongolia) in the penitentiary system, were screened at entry and twice a year [16]. However, documentation on the feasibility and evaluation of these procedures was limited [17, 18]. Contact investigations among household members and close contacts of persons with TB remained a core strategy in national TB screening policies. Nevertheless, resource limitations often disrupted adherence to the intended schedule once a person with TB was identified in a household [19]. China had the most comprehensive regular TB screening activities. Older adults received annual health screening through the Basic Public Health Service Project [20, 21] School-goers under 15 years were screened for TB infection upon admission [22] while those aged 15 and older received an annual CXR assessment [22] Health care workers were also screened annually for TB through the country’s infection prevention and control programme. Regular screening was also implemented for individuals with diabetes. While this approach was feasible, its effectiveness and economic efficiency varied depending on local TB prevalence [23].

#### Ad-hoc screening

Most screening activities in the region were conducted on an ad hoc basis. These included mobile screening units, door-to-door community visits, and hospital-based triage systems in outpatient departments. Ad hoc screening targeted high-risk groups and locations, or responded to outbreaks in settings such as schools, military camps, and prisons. However, the intensity, coverage, and frequency of these activities varied widely. Population-wide screening was shown to be effective in reducing TB prevalence and transmission [24] WHO currently recommends systematic screening for the general population in areas where TB prevalence is 0.5% or higher.^3^ While some countries like Viet Nam, Papua New Guinea, Kiribati, and the Marshall Islands had implemented demonstration projects [25–27], population-wide screening was not widely adopted elsewhere. In Viet Nam and Cambodia, screening and provision of TB preventive treatment had been integrated with ACF events [26–28]. Integration with non-communicable disease services was also advocated, aligning with Sustainable Development Goal 3.3 and the WHO End TB strategy, particularly Pillar 1 on integrated person-centred care and prevention [29–31]

### Key challenges in implementing systematic screening for TB

#### Resources (financial, workforce, logistics)

A major barrier to implementing systematic TB screening was the lack of financial resources [32] There was a particular shortfall in financial investments, especially domestic funding for TB screening activities. In countries with partial Universal Health Coverage, complex insurance schemes may only partially cover TB screening and diagnostic tests, leading to out-of-pocket expenses. In some settings, such as Lao PDR [33] CXR was not insured. The low awareness of available health insurance [34] and the administrative requirements for subsidies (e.g., enrolment and accreditation under the PhilHealth schemes in the Philippines) also limited access to screening during healthcare visits. Workforce shortages and high workloads further strained TB programmes, causing delays in diagnosis, treatment, and follow-up. These challenges were compounded by infrastructural and logistical constraints, including unreliable power supply, limited internet connectivity, and shortages of essential equipment and commodities for screening and treatment [35]

#### Awareness, acceptance, and coverage

Although most countries had TB screening guidelines, awareness among healthcare providers and implementers was often limited. This resulted in poor compliance with guidelines and directives from the National TB Programme. Public understanding of TB was also low, and pervasive stigma remained a significant barrier [36] discouraging active participation of both people at risk for or with presumptive TB in TB screening activities [35]

These factors directly affected the coverage and reach of TB screening programmes. Effective screening strategies required broad coverage, high intensity, and the use of sensitive tools [37]. Despite the growing recognition of asymptomatic TB (previously known as subclinical TB), screening in the seven surveyed countries often relied on symptom-based methods. Symptoms information was subjective and prone to recall bias, influenced by the respondent, the interviewer, and the timing of the questionnaire [38, 39]. But sensitive tools, such as CXR, were underutilised due to limited resources [40–44]

Reaching vulnerable populations, such as migrants and children, remained particularly challenging for screening and follow-up [36] Structural and geographic barriers further limited access to TB services in rural and remote areas, leaving many without essential screening and treatment [36, 45].

#### Impact of COVID-19

The COVID-19 pandemic significantly disrupted health systems and TB control efforts, reversing the decades-long decline of TB burden and mortality [46–48] TB screening and testing activities declined due to restrictions on social gatherings and movement [49] Fear of contracting COVID-19 also deterred vulnerable populations from visiting healthcare facilities for TB screening [50]. In high TB-burden countries, resources were reprioritised. GeneXpert systems were used for COVID-19 testing, TB workforce was reassigned to pandemic response, and budgets were reallocated to COVID-19-related needs [51] Despite these setbacks, the pandemic highlighted the need for robust reporting systems, improved health education and communication, service integration, and a strengthened healthcare workforce. It also underscored multi-sectoral collaboration, particularly in building community networks to reinforce the health system for pandemic preparedness and optimal TB control [52, 53]

### Country action plans to improve systematic screening for TB

Among the countries surveyed, strategic activities in the country action plans could be broadly summarised across two time horizons. In the short term, countries prioritised workforce training, strengthening health system capacity, expanding access to essential screening and diagnostic tools like CXR and molecular diagnostics, and intensifying case-finding among high-priority groups. Medium and long-term strategies focused on continuous quality improvements in screening, diagnosis, and TB management. Countries also aimed to scale up the implementation of other recommended TB screening tools that have not yet been adopted in routine practices. Common themes across countries included decentralising implementation to empower local stakeholders, mobilising financial resources, and increasing the intensity and frequency of screening efforts to achieve the end-TB goals. The following synthesis outlines key interventions based on country experiences to operationalise these plans (in no specific order).

#### Scaling up screening and diagnostics

1. Developing comprehensive guidelines to standardise screening protocols and support integration into routine care, particularly in light of the growing importance of cross-programmatic, multi-disease screening approaches.
2. Building local capacity by integrating TB-related capacity-building into existing cross-programmatic platforms, such as continuing medical education programmes, or through routine mentoring and supervisory visits.
3. Decentralising implementation, empowering local decision-making for tailored local strategies, and monitoring progress against national TB targets.
4. Using local data to prioritise high-burden populations and regions, recognising that target groups for screening are dynamic and context-specific.
5. Investing in sensitive, symptom-agnostic tests like CXR and molecular diagnostics, alongside infrastructure improvements to support their use.
6. Promote local innovations using bottom-up approaches to align interventions with community needs.

#### Community participation

1. Leveraging the WHO multi-sectoral accountability framework [54] to promote high-level engagement and cross-sectoral collaboration. Beyond legal adoption, it is essential to ensure meaningful engagement, high-level participation, and robust monitoring.
2. Integrating TB screening into broader health services to increase the efficacy and reach of screening programs.
3. Mobilising communities to raise awareness, reduce stigma, and improve screening uptake.
4. Bringing services closer to people. Contextualise activities based on local needs (e.g., conducting screenings during after-work hours or over weekends) to improve access.
5. Improving the acceptance of active case finding among both healthcare workers and at-risk populations.
6. Providing incentives for providers and patients, such as subsidies for CXR or performance-based rewards, while recognising the need to assess long-term sustainability.
7. Enhancing public education through schools and mass communication to improve disease literacy.

#### Financing, integration, and sustainability

1. Generating evidence on the effectiveness and cost-effectiveness of interventions to support scale-up.
2. Reducing reliance on external funding by promoting sustainable investment models.
3. Adopting a multi-sectoral approach by engaging implementers, policymakers, affected communities, and stakeholders across various sectors.
4. Integrating TB strategies into existing health infrastructure and leveraging current systems to maximise reach and impact.
5. Documenting and disseminating success stories from demonstration projects to inform replication and advocacy.

## Conclusion

In conclusion, this paper highlighted the diverse TB screening practices across seven high-burden countries in the Western Pacific Region. While guidelines and implementation approaches varied, all countries prioritised high-risk groups such as those living with HIV and household contacts. Innovations like digital CXR with CAD have improved detection, but challenges remain due to limited awareness, acceptance, and resources. Strengthening staff training and expanding the use of sensitive screening tools, such as CXR and molecular diagnostics, are essential next steps. Scaling up evidence-based screening and integrating TB services with broader health systems and universal health coverage will improve resilience, access, and equity. Achieving End TB goals will require stronger multi-sectoral collaborations and sustained political and financial commitment to reach the “missing millions” and reduce TB incidence and mortality.

## Data Availability

No datasets were generated or analysed during the current study.
